# Inter-Organelle Contact Sites Mediate the Intracellular Antioxidant Activity of Platinum Nanozymes: A New Perspective on Cell–Nanoparticle Interaction and Signaling

**DOI:** 10.1021/acsami.2c22375

**Published:** 2023-01-11

**Authors:** Vincenzo Migliaccio, Naym Blal, Micaela De Girolamo, Valentina Mastronardi, Federico Catalano, Ilaria Di Gregorio, Lillà Lionetti, Pier Paolo Pompa, Daniela Guarnieri

**Affiliations:** † Dipartimento di Chimica e Biologia “Adolfo Zambelli”, Università degli Studi di Salerno, Fisciano, Salerno 84084, Italy; ‡ Nanobiointeractions & Nanodiagnostics, Istituto Italiano di Tecnologia (IIT), Via Morego 30, Genova 16163, Italy; § Electron Microscopy Facility, Istituto Italiano di Tecnologia (IIT), Via Morego 30, Genova 16163, Italy

**Keywords:** platinum nanoparticles, inter-organelle contact sites, antioxidant activity, nanozymes, mitochondria, SOD2, MFN2, ROS

## Abstract

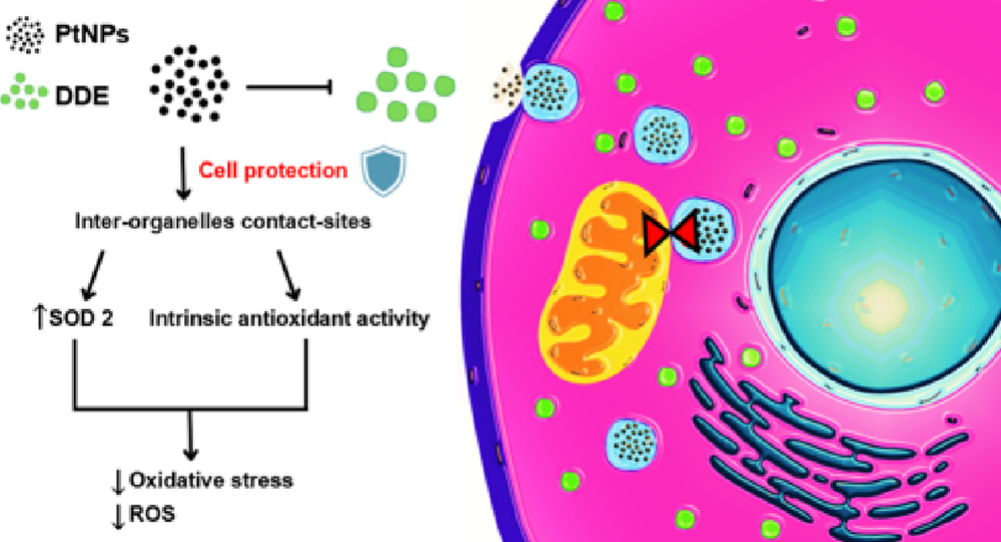

The catalytic and antioxidant properties of platinum nanoparticles (PtNPs) make them promising candidates for several applications in nanomedicine. However, an open issue, still shared among most nanomaterials, is the understanding on how internalized PtNPs, which are confined within endo-lysosomal compartments, can exert their activities. To address this problem, here we study the protective effect of 5 nm PtNPs on a human hepatic (HepG2) cell line exposed to dichlorodiphenylethylene (DDE) as a model of oxidative stress. Our results indicate that PtNPs are very efficient to reduce DDE-induced damage in HepG2 cells, in an extent that depends on DDE dose. PtNPs can contrast the unbalance of mitochondrial dynamics induced by DDE and increase the expression of the SOD2 mitochondrial enzyme that recovers cells from oxidative stress. Interestingly, in cells treated with PtNPs—alone or in combination with DDE—mitochondria form contact sites with a rough endoplasmic reticulum and endo-lysosomes containing nanoparticles. These findings indicate that the protective capability of PtNPs, through their intrinsic antioxidant properties and modulating mitochondrial functionality, is mediated by an inter-organelle crosstalk. This study sheds new light about the protective action mechanisms of PtNPs and discloses a novel nano-biointeraction mechanism at the intracellular level, modulated by inter-organelle communication and signaling.

## Introduction

The interest in platinum nanoparticles (PtNPs) is rapidly growing in the biomedical field, owing to their biocompatibility coupled to their efficient catalytic properties, making them promising artificial enzymes.^1−7^ For instance, PtNPs can catalyze the reduction of H_2_O_2_ to water and molecular oxygen mimicking the catalase (CAT) enzyme.^5^ Moreover, their activities as horseradish peroxidase (HRP)^3,8^ and superoxide dismutase (SOD) have also been described.^9^ In addition, Pt-based nanoparticles are able to absorb light in the biological window (650–850 nm), in an extent depending on their physical–chemical properties (e.g., size, shape, and crystalline structure), suggesting their employment as photosensitizers in photothermal therapy (PTT).^10−15^ Furthermore, thanks to the abovementioned multiple features, PtNPs can be designed for combination therapy in order to improve treatment strategies.^16−21^ Remarkably, the high stability of PtNPs in acidic intracellular compartments increases their cytocompatibility and tolerance in vivo compared to other metal nanoparticles,^22,23^ reducing possible adverse effects, while maintaining high catalytic efficiency in situ. To achieve PtNPs with specific properties and ad hoc sizes, two well-documented approaches exist, namely, bottom-up and top-down methods.^24,25^ In the top-down method, a large metal structure is mechanically broke down; in this way, the size distribution and morphologies are strictly controlled leading to the generation of fine nanoparticles.^26^ In the bottom-up approach,^27^ atoms and molecules are assembled to generate NPs. Examples of bottom-up methods are laser pyrolysis, nanostructural precipitation, and self-assembly of monomers/polymers. PtNPs of tailored dimensions (shape and size) are synthesized through various chemical, physical, and biological (bacteria, fungi, plants) methods.^28^ The in vitro enzyme-like properties of PtNPs offer a broad selection of potential applications in nano- and biomedicine, including preventive therapies for some types of cancer and cardiovascular diseases,^21,29^ thanks to their functional integration as nanocarriers and nanozymes. Recently, we demonstrated the capability of citrate-capped PtNPs to reduce the endogenous ROS level and their overproduction following an external oxidative insult in HeLa cells.^30^ The application of the same PtNPs as radical scavenging material was also demonstrated in a cellular model of cerebral cavernous malformation, a rare cerebrovascular oxidative stress-related disease. At low concentrations, PtNPs were able to completely restore the cellular physiological homeostasis after 48 h of treatment.^1^ Despite that these findings suggest the great potential of PtNPs for new and diverse applications, the mechanisms that PtNPs use to exert their antioxidant activity in the cells are not elucidated yet. It is known that PtNPs are internalized by cells through endocytosis and hence they are confined into endo-lysosomal compartments.^30^ Even if appropriately functionalized with specific molecules (such as cell-penetrating peptides), the probability of these nanoparticles to escape the endo-lysosomal confinement is very rare.^30,31^ Therefore, how PtNPs can play their antioxidant effect in the intracellular environment is still under debate. More in general, this issue represents a key aspect that is still unresolved and shared among most nanomaterials to better understand the nano-biointeraction mechanisms.

In this work, with the aim to clarify the antioxidant activity mechanisms of PtNPs, we used a well-established in vitro experimental model of induced oxidative stress based on human hepatic cells exposed to dichlorodiphenylethylene (DDE). DDE is an environmental pollutant deriving as a by-product of dichlorodiphenyl trichloroethane (DDT). DDT was massively utilized in agriculture as insecticide leading to an accumulation in soil, water surface, and other environmental compartments.^32,33^ Due to their physiochemical properties, DDT and DDE act as endocrine-disrupting chemicals causing hormonal and metabolic disorders.^34−36^ According to the US National Cancer Institute (NCI), the main target organ of DDE in mammalian species seems to be the liver.^37,38^ It has been reported that DDE can affect the functionality of organelles involved in metabolic detoxification processes that take place in hepatocytes, such as mitochondria.^39^ Mitochondrial bioenergetics damage, impairment of the mitochondrial respiratory chain and ATP production, and alterations of protein content and enzymatical activity of cytosolic and mitochondrial antioxidant enzymes are some of the side effects demonstrated to be associated with DDE exposure.^35,40−45^ As a result of mitochondrial dysfunction, an increment in ROS generation, a decrease in mitochondrial membrane potential, and a release of cytochrome c (cyt c) into the cytosol may occur leading to apoptosis.^46−49^ In addition, these damages at the liver level may induce insulin-resistance/diabetes onset,^50,51^ thus compromising the human health.

To test the effect of PtNPs to reduce the stress related to the exposure to DDE, in this study we used hepatocarcinoma HepG2 cells as a model of liver tissue in vitro.^52^ We carried out a systematic assessment of the effect of monodisperse, citrate-capped, 5 nm PtNPs on a HepG2 cell line with or without the co-incubation with DDE. Two doses of DDE were used, namely, 30 and 100 μM, to assess the effect of nanoparticles at sublethal and lethal concentrations of pesticide. Cell response to the different treatments was studied by analyzing several cellular parameters. Cell viability, cell morphology, and mitochondrial functionality were analyzed through different and complementary techniques to demonstrate the protective role of PtNPs against the stress induced by DDE environmental pollutants. The obtained results were correlated with the detailed ultrastructural analysis to elucidate the mechanisms of antioxidant activity of PtNPs within the intracellular environment. We focused our attention on the organelles involved in cellular response to PtNPs and oxidative stress (i.e., endo-lysosomes, mitochondria, and endoplasmic reticulum), and in particular, we analyzed the emerging mechanisms of inter-organelle interaction.

## Materials and Methods

### Materials and Reagents

p,p′-DDE (DDE) lyophilized powder was purchased from Sigma. DDE was dissolved in DMSO (Sigma) to prepare stock solutions at two different concentrations, 30 and 100 mM. Before starting each experiment, stock solutions were further diluted 1:1000 in cell culture media to prepare 30 and 100 μM DDE working solutions.

### PtNP Synthesis and Characterization

PtNPs were synthesized as previously reported.^1^ The morphology of the PtNPs was analyzed by transmission electron microscopy (TEM) (JEOL JEM-1011), operating at an accelerating voltage of 100 kV (Figure S1). Several images were collected to determine the size distribution of the NPs (*n* = 300), and ICP measurements were made to calculate NP concentrations.

### Cell Culture

Human hepatocarcinoma HepG2 cells were cultured in Minimum Essential Medium (MEM, Euroclone) supplemented with 10% (v/v) fetal bovine serum (FBS, Euroclone), 100 U mL^–1^ penicillin, and 100 mg mL^–1^ streptomycin (Euroclone). Cells were maintained in an incubator at 37 °C under a humidified controlled atmosphere and 5% CO_2_.

### MTT Assay

Cell viability was evaluated by measuring the cell metabolic activity using MTT assay (Merck-Sigma). Briefly, 1 × 10^4^ HepG2 cells suspended in 100 μL of cell culture medium were seeded in each well of a 96-well plate. After 1 day from cell seeding, cells were treated with 25 and 50 μg/mL of PtNPs alone and in combination with 30 and 100 μM DDE for 24 h. PtNP concentrations were chosen for their already proved antioxidant activity and cytocompatibility in other experimental models.^1,30,53^ Non-treated cells and cells treated with DMSO and water, at the same concentrations used to dissolve DDE and PtNPs, respectively, were used as negative controls. After 24 h of treatment, cell culture medium of each well was replaced with 100 μL of fresh medium supplemented with 5 μL of MTT reagent (1 mg/mL in PBS) and incubated for 90 min at 37 °C. Afterward, the media were removed and formazan crystals were dissolved by adding 100 μL of DMSO in each well. Absorbance was measured at 595 and 655 nm using a microplate reader (Bio-Rad), and raw data were normalized to non-treated cells (considered 100%) to calculate cell viability percentage. Experiments were performed in triplicate, and data were reported as mean ± standard deviation.

### Cell Staining

For cell spreading analysis, after treatments with PtNPs and DDE, HepG2 cells were fixed with 4% paraformaldehyde for 20 min at room temperature, permeabilized with 0.01% Triton X-100 for 5 min, and blocked with blocking buffer solution (0.5% bovine serum albumin in PBS) for 20 min. Cell nuclei were stained with Hoechst 33342 (Thermo Fisher Scientific), and actin microfilaments were localized by phalloidin red (Thermo Fisher Scientific). Cytochrome C was localized by using mouse anti-cytochrome c primary antibodies (Santa Cruz Biotechnology) and Alexa488 anti-mouse secondary antibodies (Thermo Fisher Scientific). Cells were incubated with primary antibodies in blocking buffer solution (1:200) for 1 h, and after several washes with PBS, cells were incubated with the secondary antibodies (1:500) for 30 min. Mitochondria were stained by using MitoTracker Green (Thermo Fisher Scientific) in living cells upon 24 h treatments with PtNPs and DDE. Images were acquired by an inverted epifluorescence microscope (Olympus CKX41). The analysis of the acquired images was performed by ImageJ software to measure the cell spreading area, nuclei, mitochondria network, and morphology.^54,55^

### DCF Assay

3 × 10^4^ HepG2 cells were seeded in 96-well microplates and treated with 30 and 100 μM DDE alone and in combination with 50 μg/mL PtNPs as described above. After 24 h, the dichlorofluorescein (DCF) assay was performed. Briefly, the cells were washed with Hank’s Balanced Salt Solution (HBSS) and incubated with 5 μM DCFH-DA (2′,7′-dichlorofluorescein diacetate, Sigma) in HBSS for 45 min at 37 °C. The cells were washed with HBSS, and the DCF fluorescence intensity was analyzed by fluorescence microscopy. Image analysis by ImageJ software was performed to measure relative fluorescence intensity of each sample. The results were normalized with respect to the negative controls (expressed as 100%). To verify the ROS scavenging activity of PtNPs, HepG2 cells were also treated for 30 min with 400 μM H_2_O_2_ after 24 h incubation with and without PtNPs and 45 min staining with 5 μM DCFH-DA as further controls.

### Western Blot (WB)

For WB experiments, ∼3 × 10^5^ cells were seeded in each well of a six-well plate. 24 h after seeding, cells were incubated with 50 μg/mL of PtNPs alone and in combination with 30 and 100 μM DDE for 24 h. Non-treated cells and cells treated with DMSO and water, at the same concentrations used to dissolve DDE and PtNPs, respectively, were used as negative controls. After 24 h at 37 °C, treatments were removed and the cells were washed with PBS and lysed at 4 °C with RIPA Buffer (Sigma) supplemented with protease inhibitors (Sigma). Cell lysates were centrifuged at 4 °C and 12,000 rpm for 15 min, and protein content was measured by Bradford assay. Equal amounts of total proteins (∼30 μg) were separated by 13% acrylamide/bis-acrylamide (29:1) gel and electroblotted onto the PVDF membrane (0.2 mm, Bio-Rad). The blots were blocked with TBS 5% milk for 1 h at room temperature. The membranes were incubated overnight at 4 °C with the following primary antibodies: Mfn2 (sc-100560, 1:1000; Santa Cruz Biotechnology, Dallas, Texas, USA); DRP1 (sc-3298, 1:1000; Santa Cruz Biotechnology) and SOD2 (PA5-30604, 1:1000, Thermo Scientific, Waltham, Massachusetts, USA), diluted in TBST 2% non-fatty acid milk. Mouse monoclonal anti-β actin (sc-376421, 1:1000; Santa Cruz Biotechnology) was used as a loading control guide to normalize protein levels in samples. Subsequently, blots were incubated with appropriate HRP-conjugated anti-mouse and anti-rabbit secondary antibodies (1:10,000; Bio-Rad Laboratories, Hercules, California, USA) diluted in TBST 5% non-fatty acid milk for 1 h at RT. Proteins were visualized according to the manufacturer’s instruction using an enhanced chemiluminescence (ECL) detection system (Elabscience) and revelated by using X-ray films. Protein quantification was performed by using Quantity One (Bio-Rad) program.

### Transmission Electron Microscopy

HepG2 cells were incubated with 50 μg/mL PtNPs alone and in combination with 30 μM DDE for 24 h and processed as previously reported.^30^ Briefly, after nanoparticle incubation, the cells were fixed for 1 h in 1.2% glutaraldehyde (Sigma-Aldrich) in 0.1 M sodium cacodylate buffer (pH 7.4, Sigma-Aldrich), post fixed in 1% osmium tetroxide in the same buffer, and washed six times for 10 min and then stained with 1% uranyl acetate in Milli-Q water overnight at 4 °C, followed by extensive washing in Milli-Q water. The samples were then dehydrated in an ascending EtOH series using solutions of 70, 90, and 96% and three times at 100% for 10 min each, incubated in propylene oxide (PO) three times for 20 min before incubation in a mixture of PO and EPON resin overnight, and incubated in pure EPON for 2 h and embedded by polymerizing EPON at 65 °C for 48 h. Ultra-thin sections of 70 nm were cut using a Leica Ultracut EM UC 6 Cryo-ultramicrotome. TEM images were collected with a JEOL JEM 1011 electron microscope and recorded with a 2 Mp charge-coupled device camera (Orius Gatan).

### Statistical Analysis

Quantitative data were reported as mean ± standard deviation (SD). Statistical analyses were performed using a one-way analysis of variance (ANOVA) followed by Bonferroni’s post hoc test by GraphPad Software. A *p* value <0.05 was considered statistically significant.

## Results and Discussion

### Effect of DDE and PtNP Exposure on Cell Viability and Morphology

**Figure 1:**
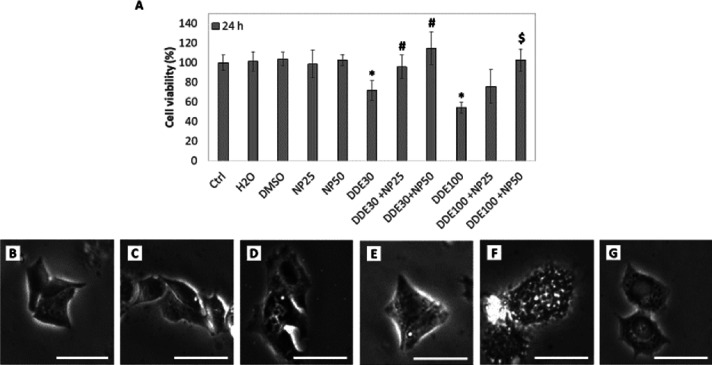
MTT assay (A) and optical microscope images of HepG2 cells non-treated (B) and after 24 h treatments with 50 μg/mL PtNPs (C), 30 μM DDE (D), 50 μg/mL PtNPs +30 μM DDE (E), 100 μM DDE (F), and 50 μg/mL PtNPs +100 μM DDE (G). Results were analyzed by one-way analysis of variance (ANOVA) (*n* = 8). *P < 0.05 vs CTRL, #*P* < 0.05 vs DDE30, $*P* < 0.05 vs DDE100. Magnification bar 20 μm.

To study the effects of PtNPs on hepatocytes exposed to DDE, we first performed an MTT assay by treating HepG2 cells with 25 and 50 μg/mL of 5 nm PtNPs and 30 and 100 μM of DDE for 24 h. Results reported in Figure 1 show that cell viability was not affected by the incubation with PtNPs, in agreement with previous data on other cell lines.^30^ Moreover, treatments with DMSO and water, used as solvents of DDE and nanoparticles, respectively, did not alter cell viability, thus excluding their possible interference in MTT results. On the other hand, incubation of HepG2 cells with DDE led to a significant decrease in cell viability after 24 h compared to non-treated control cells. In fact, for 30 μM DDE cell viability was around 80% and for 100 μM DDE it was ∼50%, in line with previously reported results.^56^ Interestingly, the adverse effect of DDE on cell viability was not observed for the cells co-incubated with PtNPs and DDE, where the cell viability percentage was significantly higher and comparable to control cells. This result was more evident for cells incubated with the highest concentration of PtNPs (50 μg/mL). Indeed, MTT results indicated 100% cell viability after 24 h co-treatment with 50 μg/mL PtNPs and both 30 and 100 μM DDE, with a recovery of about 20 and 50% with respect to samples incubated only with DDE. At 25 μg/mL, a significant recovery of cell viability was observed only for the samples treated with the lower concentration of DDE (30 μM). Likely, at 100 μM of DDE, the 25 μg/mL concentration of PtNPs was not sufficient to contrast with the pesticide effect in reducing cell viability. Therefore, we decided to use the most effective 50 μg/mL PtNP concentration for all the other experiments.

A first analysis at the optical microscope confirmed MTT observations, showing no significant alterations of cell morphology for the samples treated with PtNPs and 30 μM DDE and co-treated with PtNPs + DDE (Figure 1C,D,E,G). On the contrary, a drastic change in cell shape was evidenced after 24 h of incubation with 100 μM DDE (Figure 1F). Cells treated with the highest DDE concentration, in fact, had irregular boundaries and several vacuoles inside the cytoplasm. These observations were in line with our MTT data and previous works,^56^ confirming cell damage related to the exposure of cells to high DDE doses. It is noteworthy that the co-incubation of 100 μM DDE with 50 μg/mL PtNPs drastically reduced the effect of DDE in altering cell morphology (Figure 1G).

**Figure 2:**
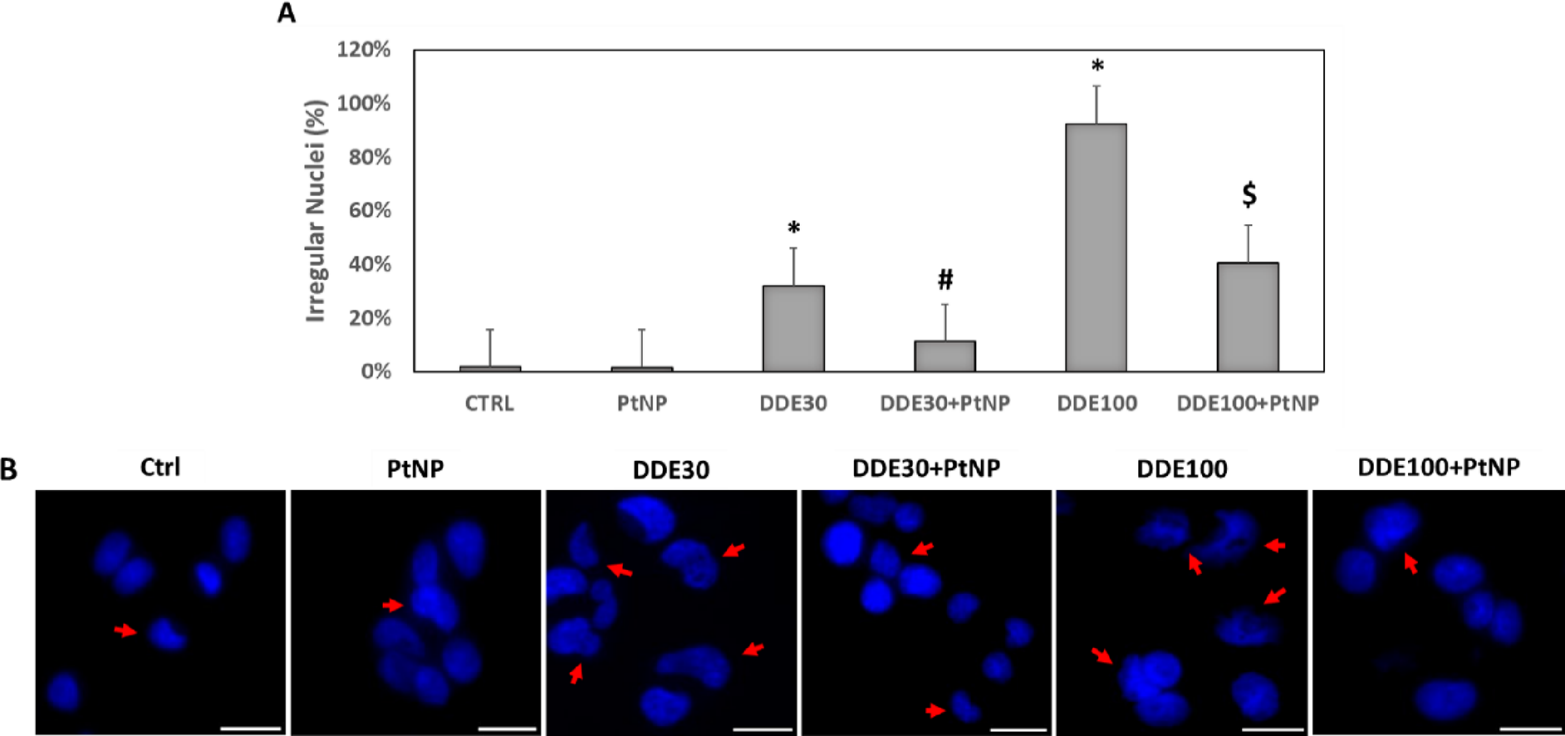
Analysis of nuclear morphology of HepG2 cells non-treated and after 24 h treatments with 50 μg/mL PtNPs, 30 μM DDE, 50 μg/mL PtNPs +30 μM DDE, 100 μM DDE, and 50 μg/mL PtNPs + 100 μM DDE (A). Data are reported as percentage of irregular nuclei and represent mean values ± standard deviation (SD). Results were analyzed by one-way analysis of variance (ANOVA) (*n* = 30). *P < 0.05 vs CTRL, #*P* < 0.05 vs DDE30, $*P* < 0.05 vs DDE100. Representative images of nuclei stained with Hoechst 33258 of HepG2 cells after treatments (B). Red arrows indicate irregular nuclei. Magnification bar 20 μm.

**Figure 3:**
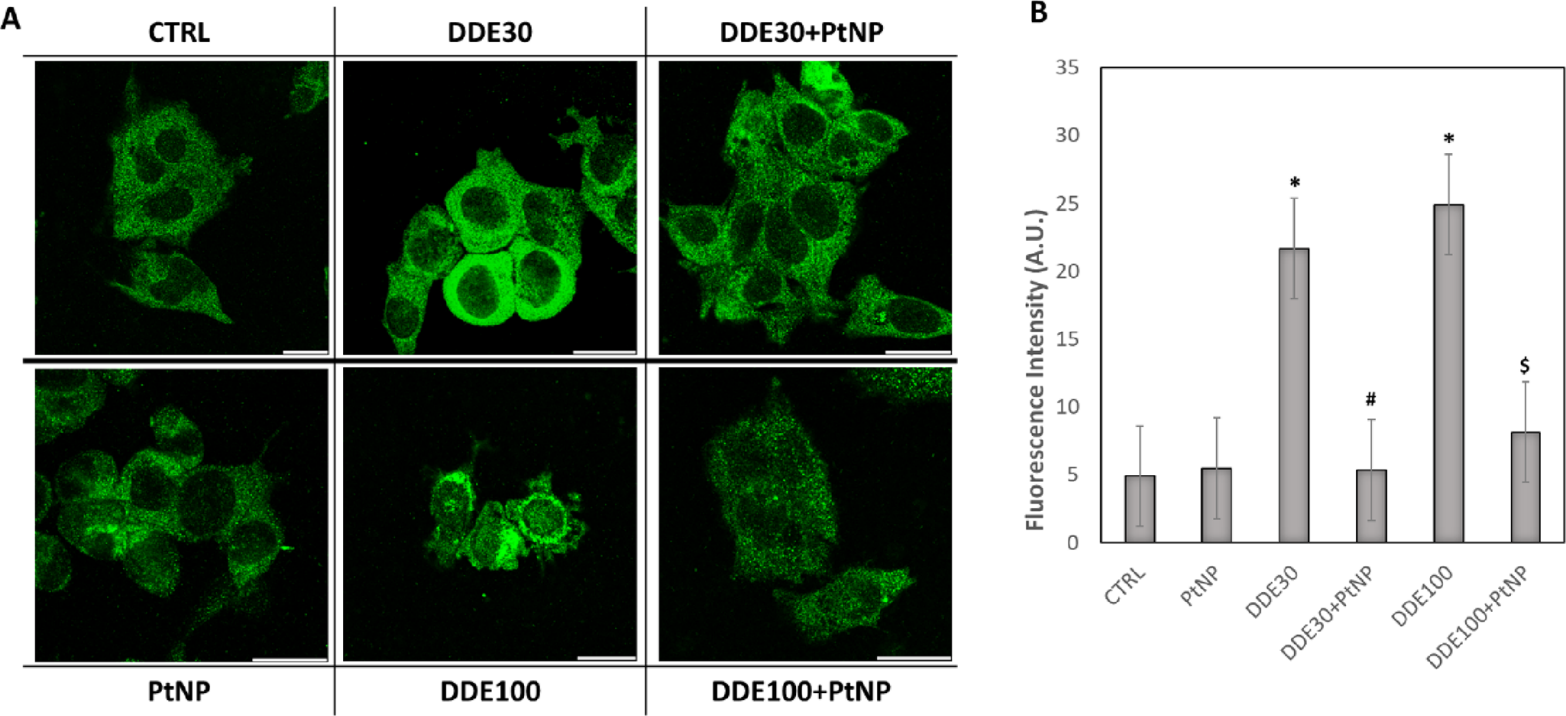
Immunolocalization of cytochrome c (green) in the HepG2 cell line before (CRTL) and after 24 h exposure to 30 μM DDE (DDE30), 100 μM DDE (DDE100), and 50 μg/mL PtNPs and co-incubation with DDE30 + PtNPs and DDE100 + PtNPs (A). Magnification bar 20 μm. Quantification of cytochrome c fluorescence intensity after the different treatments (B). Data are reported as fluorescent intensity (A.U.) and represent mean values ± standard deviation (SD). Results were analyzed by one-way analysis of variance (ANOVA) (*n* = 30). *P < 0.05 vs CTRL, #*P* < 0.05 vs DDE30, $*P* < 0.05 vs DDE100.

Furthermore, the analysis of cell nuclei indicated a high percentage of nuclei with an irregular shape and/or incomplete or absent boundaries after DDE exposure (Figure 2A). In particular, about 30% of nuclei at 30 μM DDE and almost 100% of nuclei at 100 μM DDE were abnormal (Figure 2A). These percentages were drastically reduced for the samples co-treated with DDE and PtNPs, confirming the protective effect of nanoparticles. Representative images of cell nuclei stained with Hoechst 33258 are reported in Figure 2B, and the red arrows indicate irregular nuclei. Moreover, many nuclei at 100 μM DDE appeared with condensed chromatin and vacuoles, indicating nuclear fragmentation typical of apoptotic cells (Figure S2). The immunolocalization of cytochrome c corroborated this analysis (Figure 3). Cytochrome c is a mitochondrial protein that can be released in the cytoplasm as a consequence of mitochondrial apoptosis.^57^ Confocal microscopy images showed an increase in cytochrome c expression for samples treated with 30 and 100 μM DDE (Figure 3A). On the contrary, the presence of PtNPs kept the expression levels of cytochrome c comparable to non-treated control cells (Figure 3A).

**Figure 4:**
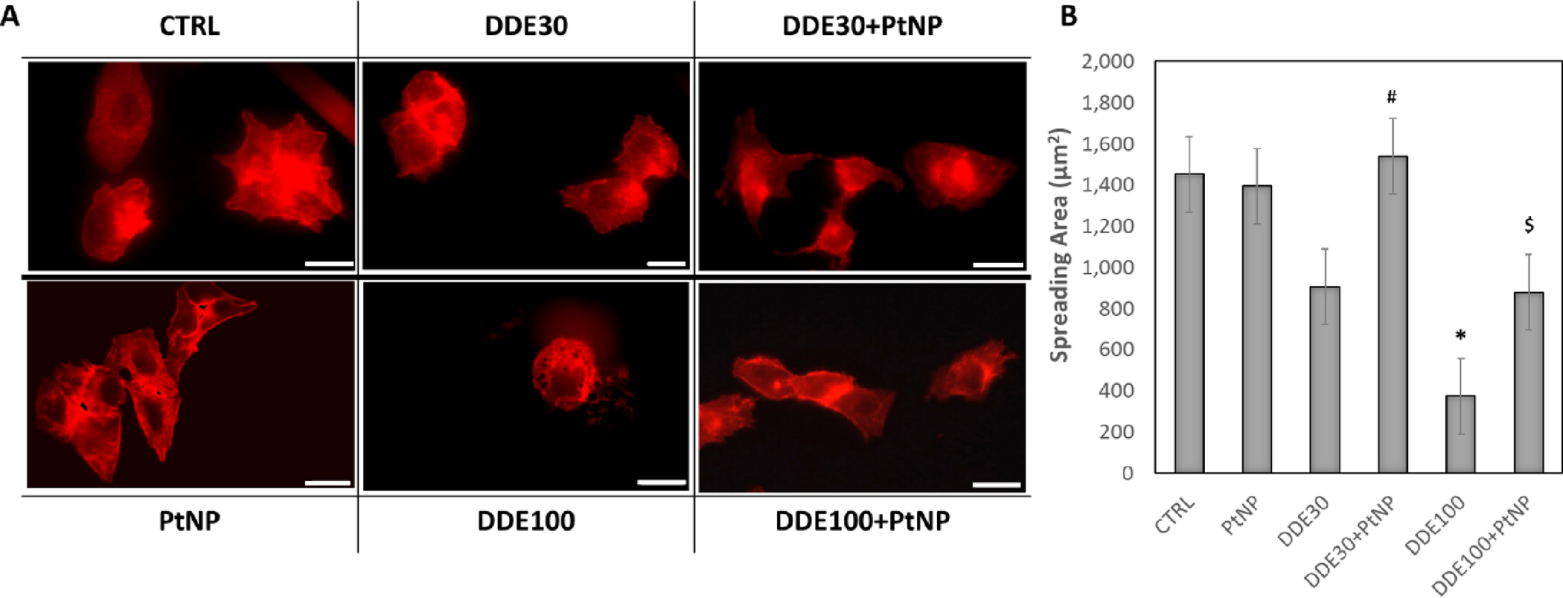
Actin microfilaments stained with red phalloidin in HepG2 cells before (CRTL) and after 24 h exposure to 30 μM DDE (DDE30), 100 μM DDE (DDE100), and 50 μg/mL PtNPs and co-incubation with DDE30 + PtNPs and DDE100 + PtNPs (A). Magnification bar 20 μm. Analysis of cell spreading area as a function of the different treatments (B). Data are reported as spreading area (μm^2^) and represent mean values ± standard deviation (SD). Results were analyzed by one-way analysis of variance (ANOVA) (*n* = 30). *P < 0.05 vs CTRL, #*P* < 0.05 vs DDE30, $*P* < 0.05 vs DDE100.

In agreement with the above results, phalloidin staining demonstrated that actin microfilaments were disassembled only upon 100 μM DDE treatment (Figure 4A), justifying changes in the cell morphology, while a correct formation of cytoskeletal fibers was observed for all the other samples (Figure 4A). As a consequence, the cell spreading area indicated a significant decrease in cell surface after treatment with 100 μM DDE (Figure 4B). A slight non-significant decrease in cell area was observed also with 30 μM DDE (Figure 4B). More interestingly, the co-incubation of DDE with PtNPs evidenced a recovery of a normal spreading area for cells treated with 30 μM DDE and a partial recovery for cells treated with 100 μM (Figure 4B). Taken altogether, these data demonstrated the ability of PtNPs to contrast/reduce the cytotoxic effect of DDE. The protective role of PtNPs depends on the DDE doses. In fact, at sublethal concentrations (i.e., 30 μM), PtNPs are able to completely reduce/inhibit DDE damages. However, it is very interesting to observe the effect of PtNPs at lethal concentrations of DDE (i.e., 100 μM) where nanoparticles protect cells, although partially.

### Antioxidant Activity at the Mitochondrial Level

**Figure 5:**
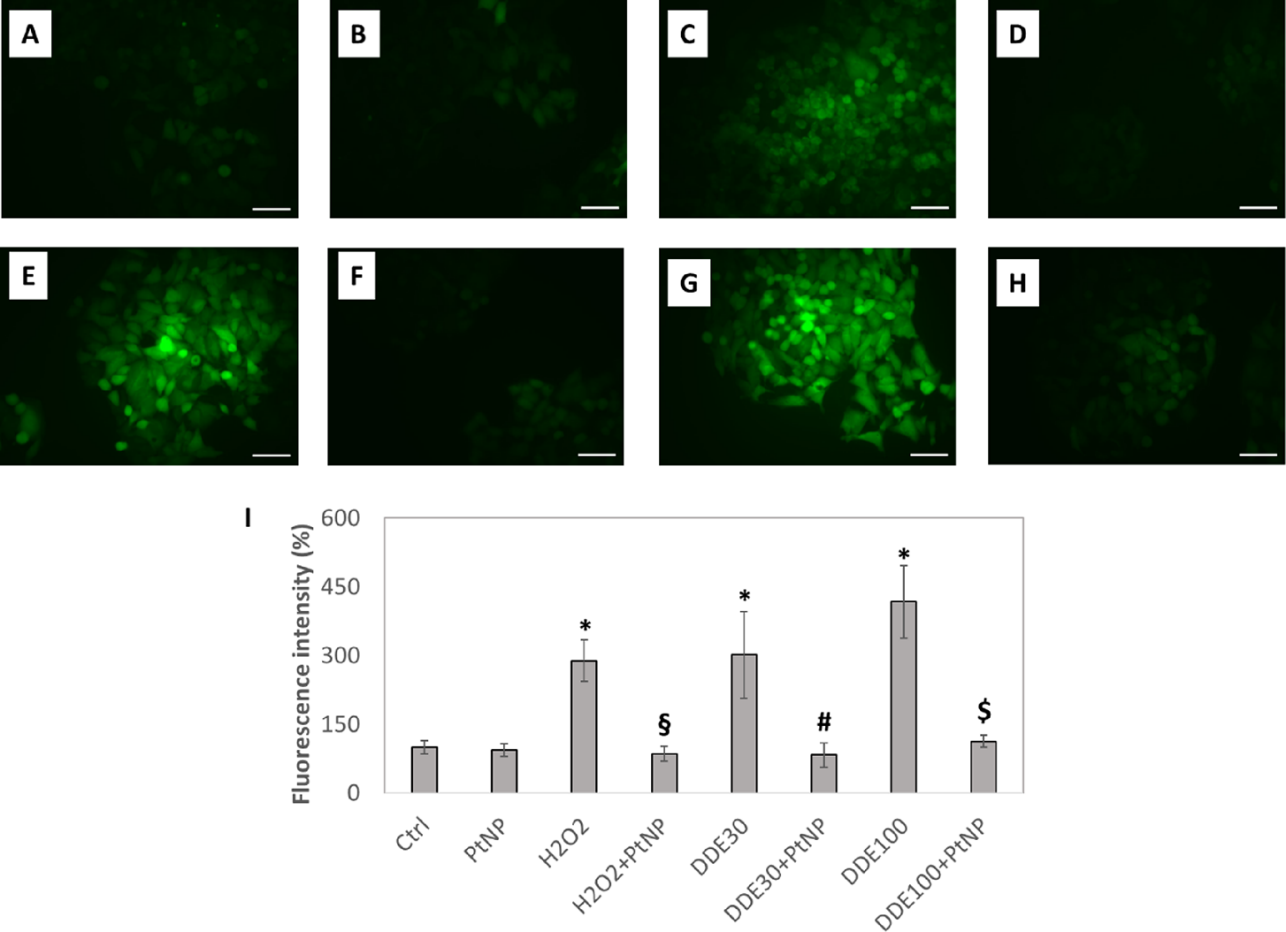
Effects of PtNPs and DDE on ROS production through DCF assay. Fluorescence images of control HepG2 cells (A) and cells treated with 50 μg/mL PtNPs (B), 400 μM H_2_O_2_ (C), 400 μM H_2_O_2_ + PtNPs (D), 30 μM DDE (E), 30 μM DDE + PtNPs (F), 100 μM DDE (G), and 100 μM DDE + PtNPs (H). Magnification bar 50 μm. ImageJ analysis of DCF fluorescence intensity as a function of the different treatments (I). Data are reported as percentage of fluorescence intensity normalized to non-treated control cells and represent mean values ± standard deviation (SD) of four biological replicates. Results were analyzed by one-way analysis of variance (ANOVA). *P < 0.05 vs Ctrl, #*P* < 0.05 vs DDE30, $*P* < 0.05 vs DDE100, §*P* < 0.05 vs H_2_O_2._

It is known that exposure to DDE induces oxidative stress causing an excessive production of reactive oxygen species (ROS).^41,48^ As we previously demonstrated in several cell types,^1,30^ PtNPs show a scavenging effect of the ROS. Therefore, we performed a DCF assay to verify the antioxidant capability of PtNPs also in the DDE-treated HepG2 cell line. DCF assay indicated an increase in ROS in HepG2 cells treated with 30 and 100 μM DDE (Figure 5E,G,I), similar to what we observed for cells treated with 400 μM H_2_O_2_, used as a positive control (Figure 5C,I). The effect of ROS generation was more evident after treatment with 100 μM DDE, showing a 4.5-fold increase compared to the control (Figure 5I). On the other hand, 30 μM DDE induced a threefold increase in ROS production (Figure 5I). Notably, PtNPs exerted their ROS scavenging effect in HepG2 cells treated with 400 μM H_2_O_2_ (Figure 5D,I) in agreement with previously reported results.^1,30^ More interestingly, the antioxidant activity of nanoparticles was observed also for cells treated with DDE (Figure 5F,H,I). In particular, ROS levels of cells co-incubated with PtNPs and DDE were similar to non-treated control cells. No increase in ROS production, compared to non-treated cells, was observed for cells incubated with PtNPs (Figure 5B,I) and DMSO (Figure S4), used as further negative controls.

**Figure 6:**
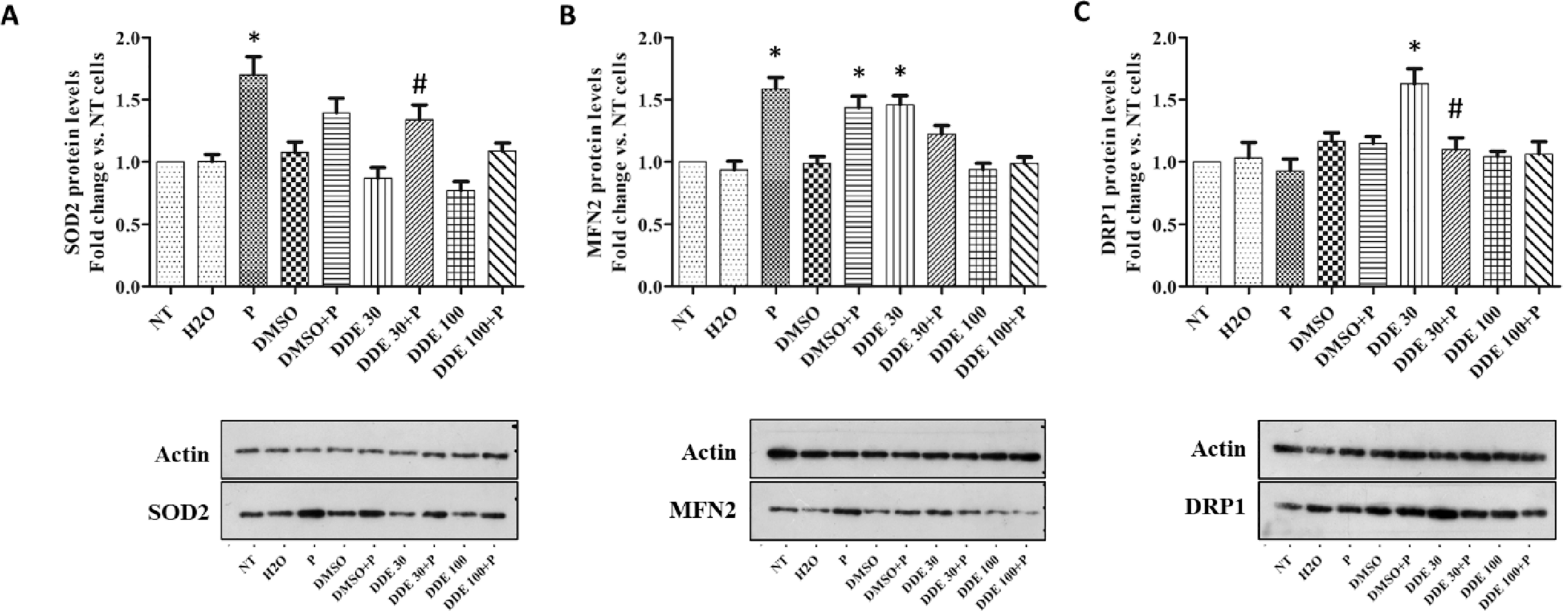
Effects of PtNPs and DDE on expression levels of mnSOD2 (A), MFN2 (B), and DRP1 (C) by WB analysis. Data are reported as fold of change of the analyzed proteins with respect to non-treated control cells and represent mean values ± standard deviation (SD) of nine biological replicates. Statistical differences were evaluated by one-way ANOVA followed by Bonferroni post hoc test. *P < 0.05 vs NT, #*P* < 0.05 vs DDE 30.

In oxidative stress conditions, most of the ROS are produced by mitochondria. Besides playing a vital role in energy production and being involved in programmed cell death, mitochondria are a cellular target susceptible to chemicals, such as DDE.^58^ Therefore, we focused our attention on these organelles with the aim to elucidate the mechanisms of protection mediated by PtNPs against DDE damage. First, we analyzed the expression of SOD2 protein to evaluate in detail the cell response to oxidative stress induced by exposure to DDE alone and in combination with PtNPs. SOD2 is a mitochondrial enzyme with antioxidant activity.^59^ As expected, a slight reduction in expression levels of SOD2 was revealed after treatment of HepG2 cells with both 30 and 100 μM DDE (Figure 6A). This result agreed with previously reported observations about the hypothesis that the pesticide affects the mitochondrial electron transport chain (ETC) causing oxidative stress and, hence, damages to mitochondria functioning.^56^ On the contrary, western blot results indicated a significant increment of SOD2 expression upon exposure of HepG2 cells to PtNPs (Figure 6A). This increment was maintained also when PtNPs were co-incubated with DDE (Figure 6A), thus contrasting the negative effect of DDE on SOD2 enzyme expression. Our observations in oxidative stress analysis by DCF assay and SOD2 expression, together with previously reported studies,^1,8,30^ suggest that PtNPs likely act at two levels: (i) directly, thanks to their intrinsic ROS scavenging activity; (ii) indirectly, by stimulating the production of SOD2 enzyme that is involved in the reduction of mitochondrial ROS. SOD2 is the most easily inducible form and can increase its levels up to 10 times in the presence of drugs and cytokines.^59^ Defects in the expression of SOD2 can cause oxidative damage to the liver, while its overexpression plays a protective role.^59^ Therefore, we demonstrated for the first time that PtNPs protect cells from DDE damage not only through their catalytic activity but also by inducing SOD2 expression. The protective property of PtNPs against oxidative stress offers numerous advantages and can be applied to develop new therapeutic strategies for several diseases such as cancer. Indeed, it is known that ROS generated by hypoxia in the tumor environment can fuel tumor growth.^60^ Therefore, inhibition of ROS generation reduces cancer progression.^61^ Moreover, PtNP antioxidant ability can be combined with their excellent photothermal properties, thus constituting a new more performing tool in the application of PTT^62^ compared to other nanomaterials with similar properties.^63−67^ Furthermore, the antioxidant action of PtNPs can be coupled to known photothermal agents, such as black phosphorus (BP), to obtain improved performance in cancer treatment.^68^

### Mitochondrial Dynamics and Morphology after Exposure to DDE and PtNPs

Mitochondria are not static organelles; rather, they can assume variable shapes and dimensions depending on the functional status and energy requirements of the cell. Mitochondria form a dynamic network through a balance between fission and fusion processes, and mitochondrial dynamics are involved in several cell activities.^69,70^ Fission is a process of division of one mitochondrion in two. It occurs during cell division to guarantee an adequate number of mitochondria among daughter cells, but it contributes also to a quality check promoting removal of damaged mitochondria and facilitating apoptosis at high levels of cellular stress.^71^ Fusion has an important role in mitigating mitochondrial stress thanks to mixing contents of partially damaged mitochondria with healthy ones, thus inducing a sort of “functional complementation”.^71^ Fusion phenomena occur, for instance, to compensate mutations and/or deletions of mtDNA producing a mix of wild-type and mutant mitochondrial genomes inside the mitochondria, avoiding/reducing the onset of mitochondrial diseases of maternal inheritance.^71^ Some stress factors can affect homeostatic balance between mitochondrial fusion and fission, leading to fusion excess through a process called “stress-induced mitochondrial hyperfusion” (SIMH).^72^ This unbalance causes a disruption of the mitochondrial network with a consequent loss of mtDNA and defects in the respiratory chain with ROS increment.^69^ Moreover, number, shape, intracellular distribution, and functions of mitochondria are fundamental to maintaining mitochondrial homeostasis in response to oxidative stress. Burgos and co-workers previously demonstrated the effect of DDE in perturbing mitochondrial dynamics in the liver.^56^ In this work, we evaluated the expression levels of MFN2 and DRP1 proteins, involved in mitochondrial fusion and fission, respectively, upon treatment with DDE and PtNPs in the HepG2 cell line. The purpose was to verify a possible relationship between fusion and fission processes as mitochondrial adaptation in response to DDE-induced oxidative stress and PtNP-protective effect. The chart reported in Figure 6B shows the cellular content of MFN2. A significant increment of MFN2 amount in cells treated with 30 μM DDE compared to control cells (NT) was observed, indicating that in these experimental conditions, the pesticide induced mitochondrial fusion. Considering the cells treated with PtNPs and PtNPs+DMSO, an increase in MFN2 expression was also revealed, suggesting that the nanoparticles are able to stimulate the mitochondrial fusion process, too. Since the fusion process occurs also to respond to an increase in energy requirements of the cells to enhance mitochondrial performance absolving a critical role in maintaining functional mitochondria for ATP production,^73^ the effect of PtNPs in MFN2 upregulation may depend on the need of the cell to produce more energy to allow particle internalization mechanisms. Furthermore, it has been recently proposed that MFN2 may regulate cellular metabolism independently of its ability to induce mitochondrial fusion.^74^ Indeed, MFN2 action in cells is not only restricted to the regulation of mitochondrial shape but also directly involved in multiple signaling pathways that include the regulation of mitochondrial metabolism, apoptosis, shape of the other organelles, and cell cycle progression.^75^ As a confirmation of the participation of MFN2 in additional cellular mechanisms, MFN2 localization has been proved not only in mitochondria but also in the rough endoplasmic reticulum (RER) membrane. De Brito and Scorrano demonstrated that MFN2 is enriched at contact sites between the RER and mitochondria; it regulates morphology of the former and directly tethers the two by means of transorganellar homotypic and heterotypic interactions.^75,76^ These contact points, named mitochondria-associated membranes (MAMs), are important to exchange nutrients, ions, and small molecules.^77−79^ In this transient contact point formation, depending on the organelles involved, a lot of protein mediators are involved including MFN2 and/or MFN1 isoforms.^79^ This suggests that the “mitochondrial kiss” represents a possible mechanism to maintain cell/organelle homeostasis in non-exacerbate stress conditions. Conversely, in our experimental conditions, cells treated with 100 μM DDE did not show significant overexpression of MFN2. Taken altogether, these data suggest that for lower concentrations of DDE (30 μM), the increment of mitochondrial fusion is induced as a cellular adaptation to oxidative stress mediated by the pesticide, because mitochondrial fusion of damaged mitochondria with healthy ones acts as a functional compensation. Probably, 100 μM DDE is a too high concentration that compromises cell functionality and, hence, hampers cell adaptation through the mitochondrial fusion process. Indeed, this hypothesis agrees with cell viability findings showing reduced viability after exposure to 100 μM DDE and 100 μM DDE + PtNPs. Furthermore, even though either PtNPs or 30 μM DDE induced an increment in MFN2 expression, cells treated with both the agents did not show a synergic effect, indicating that PtNPs and 30 μM DDE together do not provide a potentiated stimulus to fusion process at the cellular level.

Concerning DRP1 protein, a significant increase in its expression level compared to non-treated control cells was observed only at the 30 μM DDE dose (Figure 6C). This finding suggests that at such concentration, DDE also modulates mitochondrial dynamic machinery toward the mitochondrial fission process. This is likely due to a physiological adaptation that stimulates the cells to produce new mitochondria, according to the mitochondrial biogenesis process, or to eliminate parts of damaged organelles in induced oxidative stress conditions by the sublethal dose of pesticide. On the contrary, the co-incubation of 30 μM DDE with PtNPs did not increase DRP1 expression. PtNPs likely reduce the cytotoxic effect of the pesticide, shifting the dynamic equilibrium toward mitochondrial fusion by inhibiting DDE-induced fission. As observed for MFN2, 100 μM DDE, with and without PtNPs, did not change DRP1 levels compared to control.

**Figure 7:**
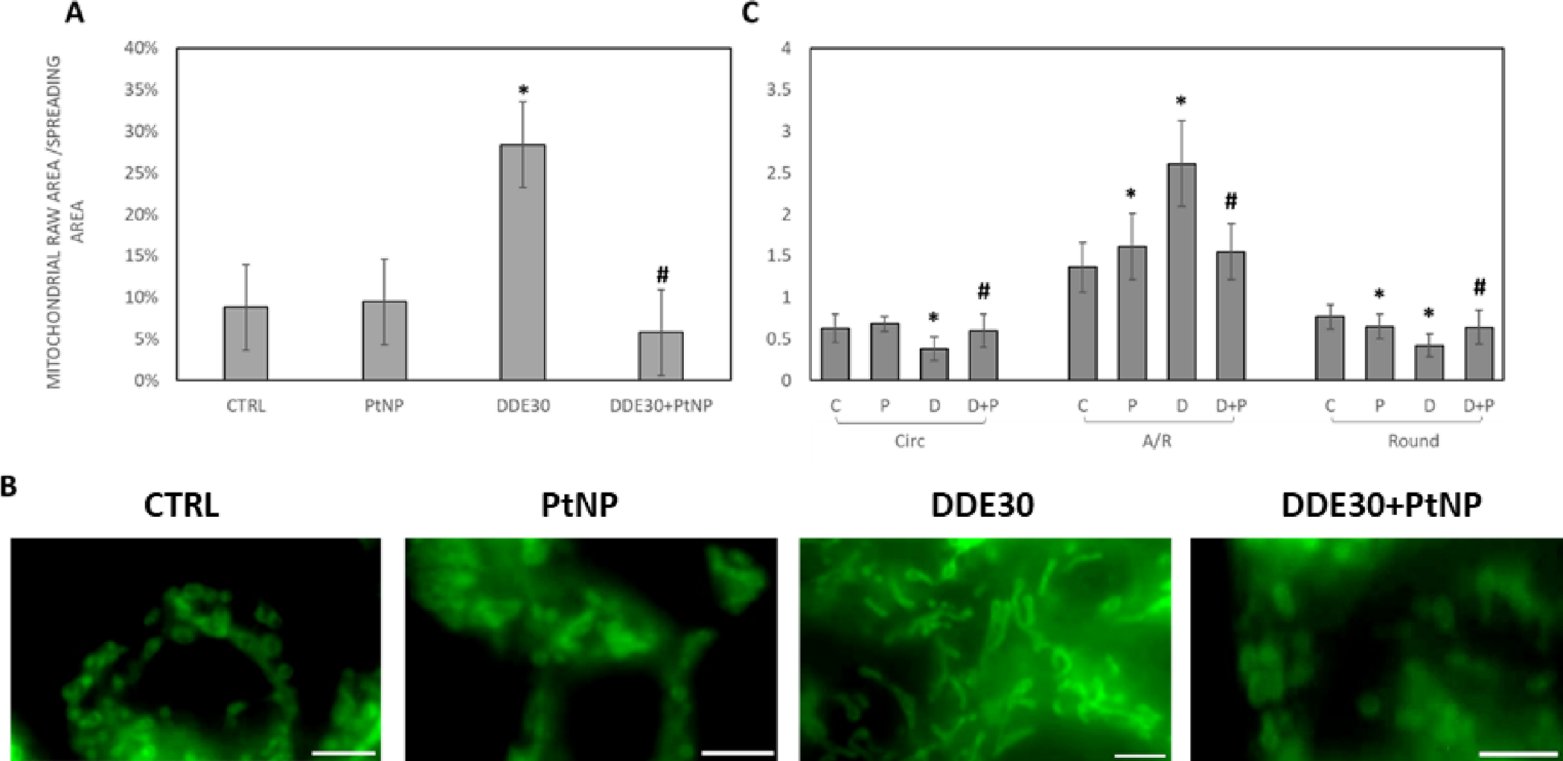
Percentage of mitochondrial area normalized to cell spreading area (A). Zoomed and cropped images representative of different mitochondrial shapes in HepG2 cells stained with MitoTracker Green after 24 h treatments with 50 μg/mL PtNPs (PtNP), 30 μM DDE (DDE30), 50 μg/mL PtNPs +30 μM DDE (DDE30 + NP) (magnification bar: 5 μm) (B). Analysis of mitochondrial morphology as a function of the different treatments (C). Mitochondrial morphology was expressed according to three shape descriptors, namely, circularity (Circ), aspect ratio (A/R), and roundness (Round) calculated with ImageJ software.^54,55^ Results were analyzed by one-way analysis of variance (ANOVA) (*n* = 20). *P < 0.05 vs CTRL, #*P* < 0.05 vs DDE30.

To shed light on the effect of PtNPs in protecting mitochondria from DDE-induced damage, a morphological analysis of these organelles was carried out. Since 100 μM DDE was a too high/lethal dose for HepG2 cells that did not permit to appreciate cellular adaptation to oxidative stress, we performed imaging analysis treating cells only with 30 μM DDE where adaptation was observable. Fluorescence images of mitochondria stained with MitoTracker Green in HepG2 cells showed that mitochondria were homogeneously distributed in the cytoplasm with a higher concentration/localization around the nucleus (Figure S5). No evident differences in intracellular distribution of mitochondria were visible for the different samples (Figure S5). However, the total mitochondrial area was significantly higher for 30 μM DDE samples (Figure 7A), indicating, indirectly, an increment in mitochondrial number because of pesticide exposure. This finding suggested an induction of mitogenesis mediated by the lowest dose of DDE tested in this study. Always in line with previous observations, the co-incubation with PtNPs and DDE resulted in an unaltered number of mitochondria (Figure 7A).

Analyzing mitochondrial morphology, in control HepG2 cells, mitochondria were assumed to have variable shapes, i.e., round, elongated, and, mostly, “donut” shape (Figure 7B and Figure S5). A similar behavior was observed also for cells treated with PtNPs (Figure 7B and Figure S5). Conversely, when cells were treated with 30 μM DDE, the majority of mitochondria were assumed to have an elongated shape as confirmed by the analysis of shape descriptors (Figure 7C). More precisely, the aspect ratio (A/R) of the mitochondria of cells treated with 30 μM DDE was 2.61 ± 0.52 while their circularity and roundness were 0.38 ± 0.14 and 0.42 ± 0.14, respectively. On the contrary, the shape descriptor values of control cells were 1.36 ± 0.30 (A/R), 0.62 ± 0.17 (Circ), and 0.76 ± 0.15 (Round). For cells treated with PtNPs alone and co-incubated with 30 μM DDE, the values of shape descriptors were closer to control cells, confirming again the protective effect of PtNPs in contrasting DDE damage. Moreover, the absence of elongated mitochondria after treatment with PtNPs alone was in line with the hypothesis that the increase in MFN2 expression upon exposure to nanoparticles alone depended on the involvement of MFN2 proteins in other cellular mechanisms, as discussed above.

**Figure 8:**
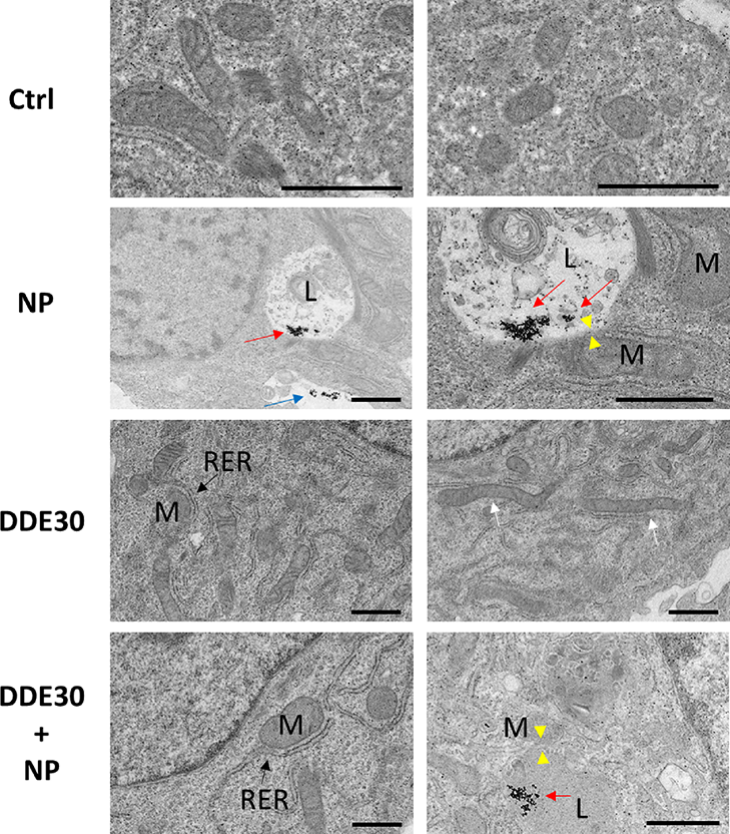
TEM micrographs of HepG2 cells non-treated (Ctrl) and after 24 h treatments with 50 μg/mL PtNPs (NP), 30 μM DDE (DDE30), and 50 μg/mL PtNPs +30 μM DDE (DDE30 + NP). Blue arrow indicates nanoparticles that are approaching the cell membrane before cellular uptake. Red arrows indicate internalized platinum nanoparticles confined in the endo-lysosomal compartment (L) upon endocytosis. Yellow arrowheads highlight the contact sites between lysosomes (L) and mitochondria (M). White arrows indicate elongated mitochondria. Black arrow indicates the contact sites between the rough endoplasmic reticulum (RER) and mitochondria (M).

An ultrastructural analysis of mitochondria was also performed to better elucidate the effect of the different treatments on mitochondrial morphology and to verify any possible interaction between these intracellular organelles and the internalized PtNPs. TEM analysis shows the normal distribution and morphology of mitochondria in non-treated control cells (Figure 8). For cells incubated with PtNPs, it was possible to observe nanoparticles in proximity of the cell membrane (Figure 8, blue arrow), likely entering by endocytosis. In fact, some PtNPs were visible inside the cells confined in endo-lysosomal compartments (Figure 8, red arrows). These results were similar to other previous reports for other cell lines, indicating that PtNPs are internalized by endocytosis.^30^ Mitochondrial morphology was regular and not affected by the treatment of the cells with PtNPs compared to control cells (Figure 8), in agreement with the MitoTracker analysis. Moreover, after incubation with PtNPs, TEM micrographs did not show PtNPs inside or associated with the mitochondria (Figure 8), indicating that there was no direct interaction between the internalized PtNPs and mitochondria. On the contrary, the ultrastructure of HepG2 cells changed after incubation with 30 μM DDE. Actually, we observed a higher number of mitochondria showing a more elongated shape, suggesting fusion phenomena in line with the above results (Figure 7). On the other hand, co-incubation with DDE and PtNPs maintained the regular shape and number of mitochondria. Moreover, in the presence of DDE alone or in combination with PtNPs, mitochondria were closer to RER suggesting an increase in protein synthesis and/or molecule exchange between organelles.

Since TEM micrographs showed PtNP localization in the endo-lysosomal compartments and not in the mitochondria (Figure 8), we concluded that the mitochondrial network did not directly interact with the nanoparticles. Therefore, the effect observed on mitochondrial protein up-regulation, especially concerning SOD2, may depend in part on the possible interaction of these two organelles through the formation of mitochondria–lysosome contact sites, as suggested by the overexpression of MFN2 induced by PtNP treatment (Figure 6). In fact, as reported for ER–mitochondria contact sites, also the interaction between lysosomes and mitochondria sees the involvement of MFN2.^80,81^ We hypothesized that the formation of contact sites can be responsible for the communication/signaling activity between organelles, allowing the demonstrated antioxidant performance of these nanoparticles. Interestingly, TEM micrographs of HepG2 cells treated with PtNPs and PtNPs + DDE showed some endo-lysosomal structures in close proximity to mitochondrial membranes (Figure 8), suggesting the formation of (transient) lysosome–mitochondria contact sites. To corroborate our hypothesis, we observed the formation of these inter-organelle contacts also in HeLa cells incubated with 2 and 5 nm PtNPs (Figure S6), suggesting a possible generalized mechanism of action of PtNPs. As far as we know, the intracellular mechanism described herein, underlying the direct/indirect interactions between nanoparticles and cellular organelles, is the first preliminary evidence of a novel nano-bio crosstalk that may influence cell response and functions upon nanoparticle uptake. Such nano-bio interaction mechanism might pave the way to new and interesting questions about the capability of nanomaterials to interact with and modulate intracellular machinery. However, further investigations are necessary in order to clearly elucidate the detailed cellular and molecular mechanisms involved in such processes.

## Conclusions

In this work, we analyzed the antioxidant mechanism of action of PtNPs by studying their protective role against the environmental pollutant DDE. First, we tested the capability of PtNPs to counteract the harmful action induced by DDE, leading to an adaptation of the cell that allows the persistence of the physiological cell morphology. In particular, the presence of PtNPs prevents cell death, nuclear fragmentation, and cytoskeletal disassembly. Second, we confirmed the effective antioxidant activity of PtNPs contrasting the ROS generated upon DDE exposure. Third and more interestingly, we demonstrated for the first time the capability of PtNPs to act at the mitochondrial level by promoting the expression of the SOD2 antioxidant enzyme that in a synergic mode helps to decrease the oxidative stress elicited by DDE, despite nanoparticle confinement in the endo-lysosomal compartments. In addition, PtNPs increased and rebalanced the processes of mitochondrial dynamics and mitochondrial biogenesis, hindering mitochondrial apoptosis. The protective effect of PtNPs was evident at 30 μM DDE treatment that allows to appreciate the adaptation and remodeling capabilities of the cell in a sub-lethal state; the beneficial effect of PtNPs was particularly apparent in treatments with 100 μM DDE, although the vitality, in this latter case, was not recovered. Moreover, TEM analysis revealed a direct interaction between PtNP-containing endo-lysosomes and mitochondria, suggesting that the formation of inter-organelle contact sites can be actively involved in regulating the mechanisms of action of PtNPs within the cells. These findings are of particular interest for future applications of PtNPs, as well as other nanomaterials, in the biomedical field. We believe that this study opens new questions about the mechanisms of communication/signaling inside the cells mediated by NPs. As a future perspective, understanding how to modulate the formation of inter-organelle contacts can help to improve the therapeutic performance of PtNPs as well as of other nanomaterials with similar properties.

## Supporting Information Available

TEM images and size distribution of 5 nm PtNPs; additional images of nuclei, microfilament, and mitochondria staining in HepG2 cells; additional images of DCF assay; TEM micrographs of HeLa cells after incubation with PtNPs (PDF)
