# Supplementary material for: Inter-Organelle Contact Sites Mediate the Intracellular Antioxidant Activity of Platinum Nanozymes: A New Perspective on Cell–Nanoparticle Interaction and Signaling
Source: ACS Appl Mater Interfaces. 2023 Jan 11;15(3):3882–93. doi: 10.1021/acsami.2c22375 (PMC9880958; doi:10.1021/acsami.2c22375)
Supplement: Supplementary file 1 — am2c22375_si_001.pdf [file am2c22375_si_001.pdf]

Supporting information

Inter-organelle contact sites mediate the intracellular antioxidant activity of platinum nanozymes: a new perspective in cell-nanoparticle interaction and signalling

Vincenzo Migliaccio[1,$], Naym Blal[1,$], Micaela De Girolamo[1], Valentina Mastronardi[2], Federico Catalano[3], Ilaria Di Gregorio[1], Lillà Lionetti[1,*], Pier Paolo Pompa[2,*], Daniela Guarnieri[1,*]

[1]Dipartimento di Chimica e Biologia "Adolfo Zambelli", Università degli Studi di Salerno, 84084 Fisciano, Salerno, Italy;

[2]Nanobiointeractions & Nanodiagnostics, Istituto Italiano di Tecnologia (IIT), Via Morego, 30–16163 Genova, Italy;

[3]Electron Microscopy Facility, Istituto Italiano di Tecnologia (IIT), Via Morego 30–16163 Genova, Italy

[$]These authors equally contributed to this work.

*Corresponding authors: dguarnieri@unisa.it; pierpaolo.pompa@iit.it; llionetti@unisa.it

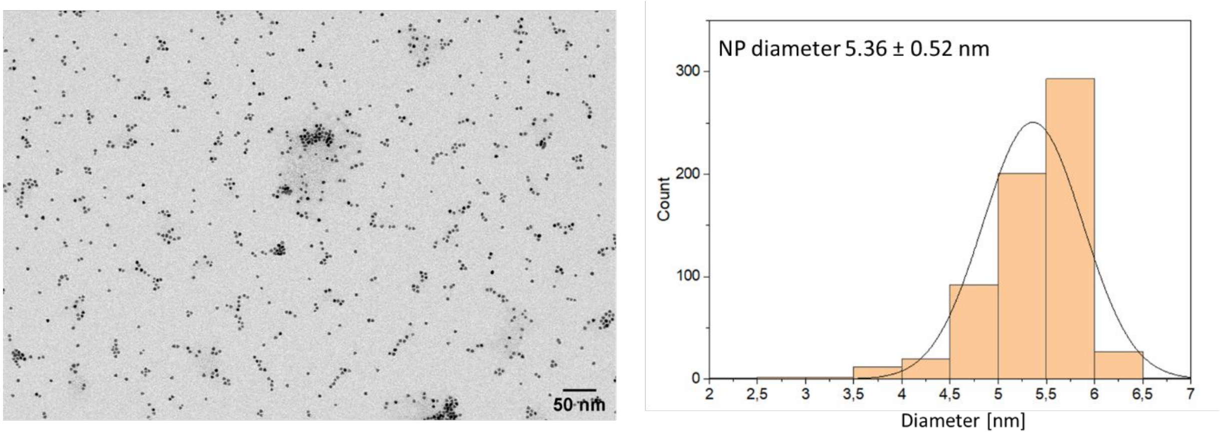

Figure S1. Representative transmission electron microscopy image of 5 nm PtNPs used in this work (left panel). Size distribution of PtNPs calculated by TEM image analysis (right panel). The mean diameter ± standard deviation (SD) of NP size is reported in the chart.

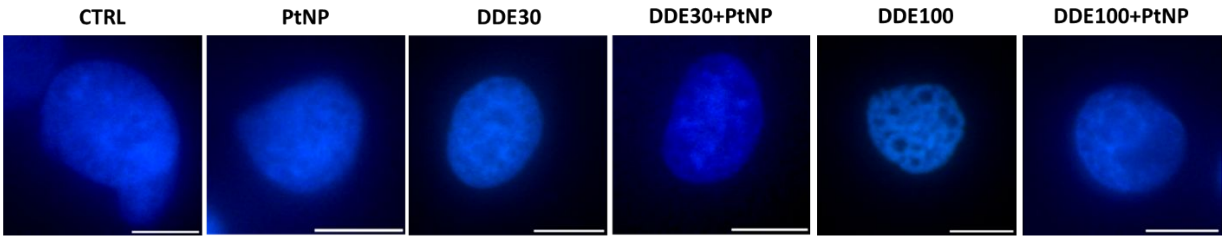

Figure S2. Representative images of cell nuclei of HepG2 cells after treatments with DDE and PtNPs. Magnification bar 10 µm.

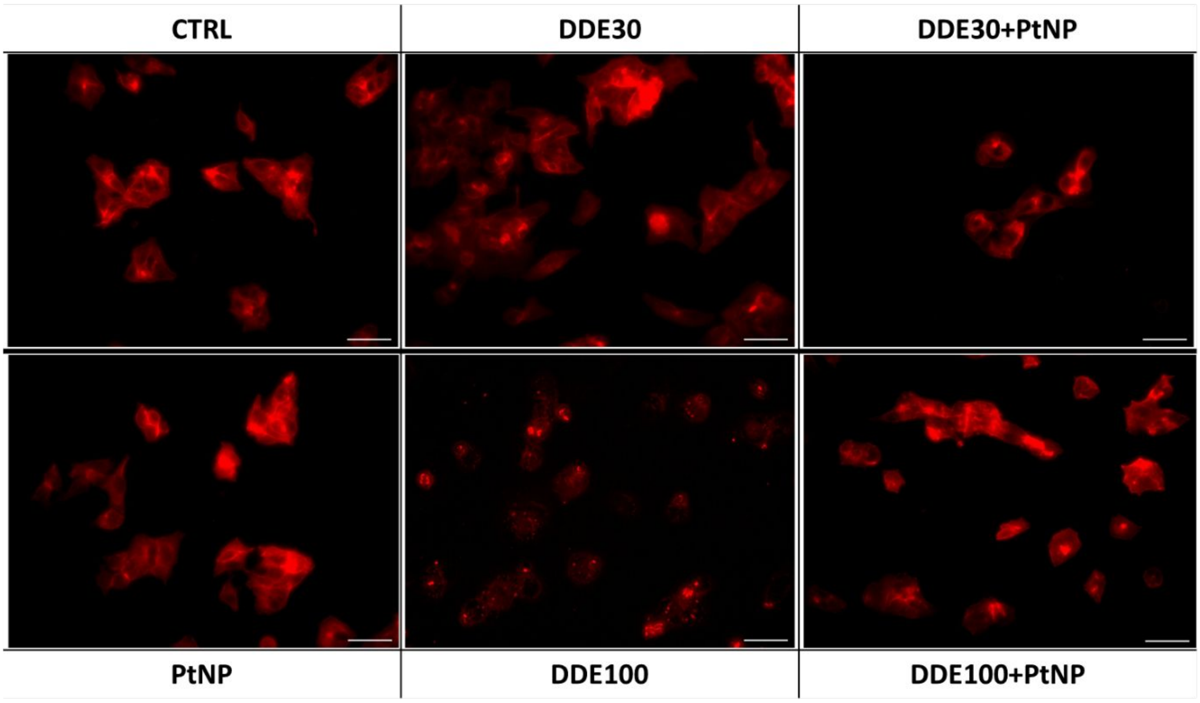

Figure S3. Representative images of actin microfilaments stained with Phalloidin red of HepG2 cells after treatments with DDE and PtNPs used to measure spreading area. Magnification bar 50 µm.

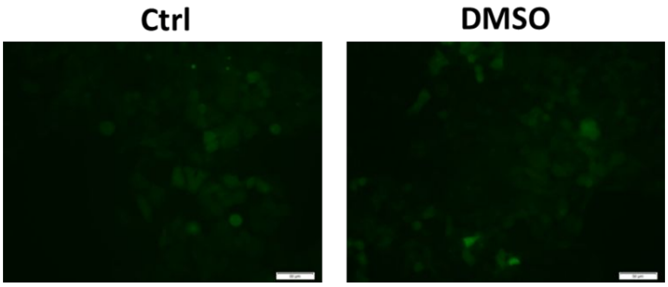

Figure S4. DCF staining of HepG2 cells non-treated and treated with DMSO. Magnification bar 50 μm.

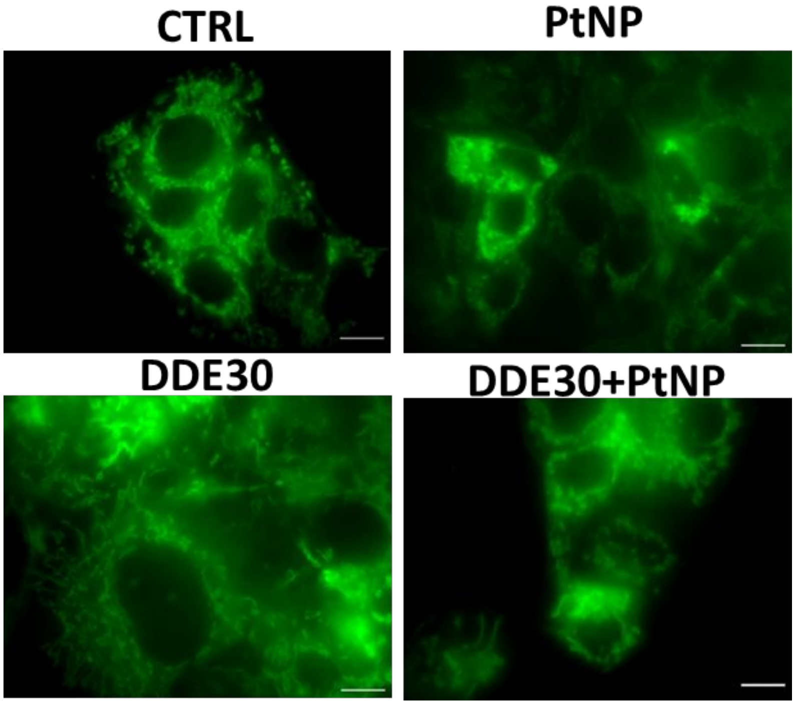

Figure S5. Fluorescence images of mitochondria stained with MitoTracker Green in HepG2 cells non-treated (Ctrl) and after 24 h treatments with 50 µg/ml PtNPs (PtNP), 30 µM DDE (DDE30), 50 µg/ml PtNPs + 30 µM DDE (DDE30+NP). Magnification bar 10 µm.

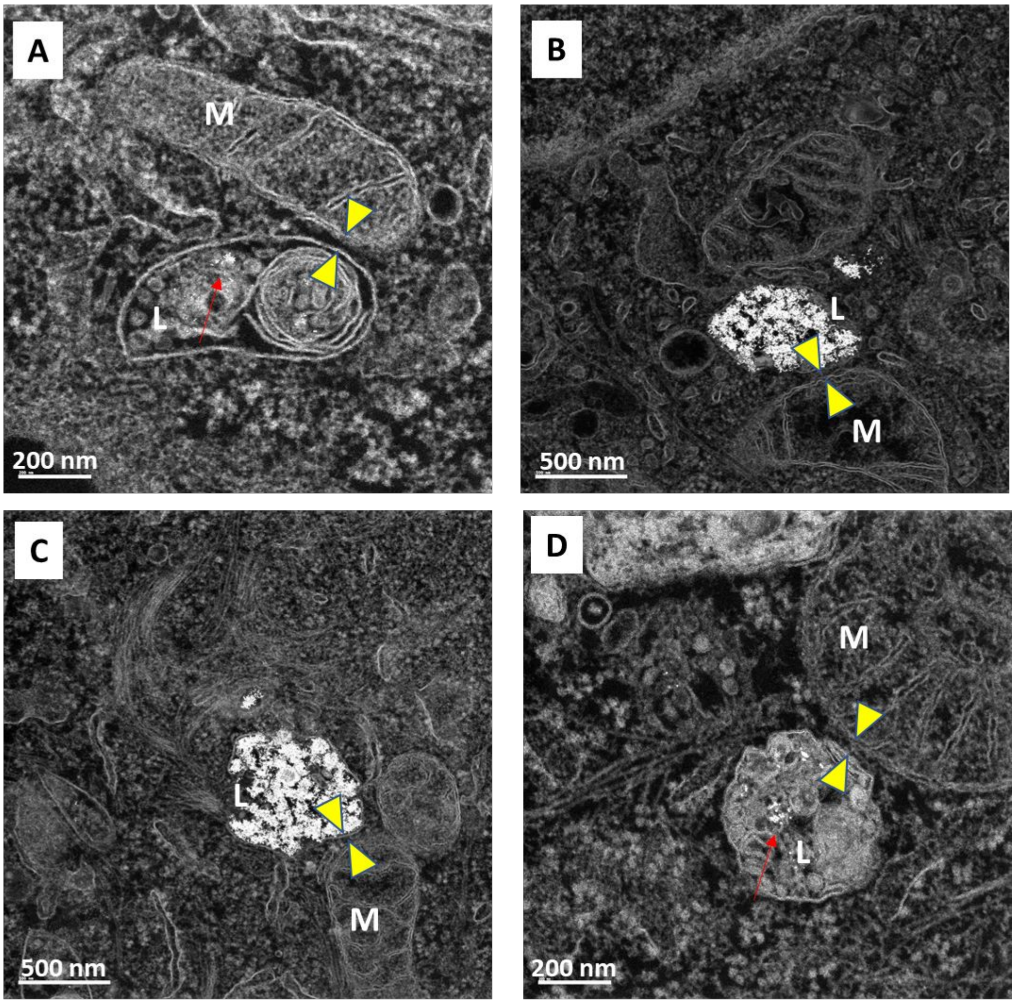

Figure S6. TEM micrographs of HeLa cells after 24 h treatments with 50 µg/ml PtNPs (NP) of 2 nm (A-C) and 5 nm (D) diameters. Red arrows indicate internalized platinum nanoparticles confined in endolysosomal compartment (L) upon endocytosis. Yellow arrowheads highlight the contact sites between lysosomes (L) and mitochondria (M).
